# Epidemiological relevant effect biomarkers for thyroid hormone system related adverse outcome pathways: a literature review

**DOI:** 10.3389/fphar.2026.1760820

**Published:** 2026-03-04

**Authors:** Maria Wielsøe, Manhai Long, Antonios K. Stratidakis, Elisavet Renieri, Dimosthenis A. Sarigiannis, Eva Cecilie Bonefeld-Jørgensen

**Affiliations:** 1 Centre for Arctic Health and Molecular Epidemiology, Department of Public Health, Aarhus University, Aarhus, Denmark; 2 Science, Technology and Society Department, Environmental Health Engineering, University School for Advanced Study (IUSS), Pavia, Italy; 3 Environmental Engineering Laboratory, Department of Chemical Engineering, Aristotle University of Thessaloniki, Thessaloniki, Greece; 4 HERACLES Research Center on the Exposome and Health, Center for Interdisciplinary Research and Innovation, Aristotle University of Thessaloniki, Thessaloniki, Greece; 5 National Hellenic Research Foundation, Athens, Greece; 6 Greenland Centre for Health Research, University of Greenland, Nuuk, Greenland

**Keywords:** adverse outcome pathway, human health, kidney toxicity, neurological outcome, thyroid follicular cancer, thyroid hormone

## Abstract

**Background:**

Many factors, such as lifestyle, medication, and environmental exposures, are reported to cause thyroid hormone system disruption (THSD) in humans, however studies linking THSD to health effects are sparse. Adverse Outcome Pathways (AOPs) provide mechanistic links from molecular events to adverse outcomes, with effect biomarkers serving as a tool to empirically anchor key events and health effects and to assess biological relevance.

**Aim:**

This review aims to identify and evaluate effect biomarkers for thyroid hormone system-related AOPs for further validation in experimental and epidemiological studies.

**Methods:**

Using AOP-wiki, we extracted and analysed thyroid-related AOPs, focusing on the eleven AOPs with mammalian evidence. We did systematic literature search to identify potential effect biomarkers for future epidemiological studies.

**Results:**

In an AOP network analysis of the eleven thyroid-related AOPs, we identified four AOP clusters, including hippocampal alterations, impaired learning and memory, thyroid follicular cell adenomas/carcinomas, and kidney toxicity. For the clusters on hippocampal alterations and impaired learning and memory, brain-derived neurotrophic factor emerged as a promising effect biomarker. For the cluster on thyroid follicular cell adenomas/carcinomas, no promising effect biomarkers with high specificity were identified, but interleukin-34, oxidative stress, and expression of several genes were found to be related to the adverse outcome. For kidney toxicity, a panel of effect biomarkers were identified, such as clusterin, cystatin-C, kidney injury molecule-1, N-acetyl-beta-d-glucosaminidase, neutrophil gelatinase-associated lipocalin, and osteopontin.

**Conclusion:**

This review operationalizes the AOP framework to support the use of mechanistically anchored effect biomarkers in human studies on THSD. By aligning key biological events with measurable endpoints, human matrices, and feasibility considerations, it provides a scientifically grounded path from mechanistic understanding to population research application. This enables more targeted biomonitoring, strengthens interpretation of epidemiological findings, and informs research and regulatory priorities for future validation efforts.

## 1 Introduction

Thyroid hormones are crucial for normal development and necessary for the proper functioning of physiological systems. Several factors can cause thyroid hormone system disruption (THSD) and many studies have investigated the effects of different exposures on thyroid hormone levels in humans (Babić et al., 2021). In 2021, Babić Leko found more than 25 studies reporting effects of smoking on thyroid-stimulating hormone (TSH), thyroid hormone and thyroglobulin levels, as well as more than 100 studies reporting effects of environmental pollutants, such as heavy metals, persistent organic pollutants, and plasticizers (Babić et al., 2021). Furthermore, several drugs or drug classes, such as amiodarone, glucocorticoids, and antiepileptic agents, can have unintended effects and cause THSD at multiple levels (Burch, 2019). Fewer studies have documented adverse outcomes of the THSD in humans, which may be due to difficulties measuring/assessing the downstream health effects in epidemiological studies.

The Adverse Outcome Pathway (AOP) framework offers a structured approach to understand the progression from a molecular initiating event (MIE), through a number of linked key events (KEs) to an adverse outcome (AO) (Ankley and Edwards, 2018). In the context of THSD, multiple AOPs have been/are developed across various species, generating a comprehensive cross-species AOP network (Haigis et al., 2023). This network serves as an evidence-based foundation for extrapolating data on THSD across species.

The increasing recognition of AOPs linking molecular-level perturbations to health (AOs) has advanced the identification of effect biomarkers (EBMs) (Haigis et al., 2023). EBMs, defined by the World Health Organization as measurable biochemical, physiological, behavioural, or other alterations associated with potential health impairments, play a pivotal role in human biomonitoring (HBM) and risk assessment frameworks (Rodríguez-Carrillo et al., 2022; Mustieles et al., 2020). These EBMs can offer insights into early biological effects, linking exposure and disease, as they cover a spectrum from initial biological changes, such as enzyme induction responses, to modifications in structure and function like phosphorylation and glycosylation which can alter protein stability and activity (Ladeira and Viegas, 2016). Additionally, EBMs can refine the risk assessment of specific chemical families and exposure to chemical mixtures, by establishing dose-response relationships, exploring mechanisms, and enhancing the biological plausibility of epidemiological associations.

The H2020 European Human Biomonitoring Initiative (HBM4EU) project started a systematic identification, validation, and implementation of EBMs in epidemiological studies to enhance the understanding of exposure-effect relationships. Leveraging the AOP framework, HBM4EU demonstrated that EBMs are not only integral to assessment of health risks but also in bridging gaps between mechanistic toxicology and epidemiological research (Fernández et al., 2022; Zare et al., 2021; Rodríguez-Carrillo et al., 2023a). In the European Union’s Horizon Europe project Partnership for the Assessment of Risks from Chemicals (PARC), the effect marker group under Task 5.3.2 has continued this work on identifying and developing EBMs based on AOPs.

In the present work, we focus on thyroid hormone-related AOPs currently listed in the AOP-Wiki. Other thyroid-related health effects not yet listed in AOP-wiki are outside the scope, such as cardiovascular disease, cardiac arrhythmias, osteoporosis, and lipid metabolism (Kortenkamp et al., 2020; Piantanida et al., 2020). This paper reflects a collaborative effort between researchers working on endocrine disruption and neurotoxicity, highlighting the shared mechanistic pathways and KEs.

To support the translation of mechanistic knowledge into population-based research, this review integrates the AOP framework with current evidence on EBMs relevant to THSD. By aligning KEs and AOs with measurable biomarkers and evaluating their feasibility in human studies, we provide a structured basis for selecting mechanistically informed endpoints. This synthesis highlights biomarkers that may already be suitable for use in epidemiological settings and identifies where additional validation would strengthen their application. Overall, this work aims to facilitate the effective incorporation of AOP-anchored EBMs into study design and public-health research on thyroid-related health effects.

## 2 Methods

### 2.1 Identification of thyroid-related AOPs

We queried the AOP-wiki (https://aopwiki.org/) in April 2023, to identify AOPs relevant to THSD following the general extraction logic described by Haigis et al. (Haigis et al., 2023).

All AOPs involving thyroid hormone synthesis, metabolism, transport, signaling, or thyroid hormone–dependent biological processes were screened. AOPs were retained if their taxonomic applicability included mammals (human and/or rodents) given the objective of identifying effect biomarkers (EBMs) relevant for human epidemiology. For each AOP, we extracted the molecular initiating events (MIEs), key events (KEs), KE relationships (KERs), and adverse outcomes (AOs).

**FIGURE 1:**
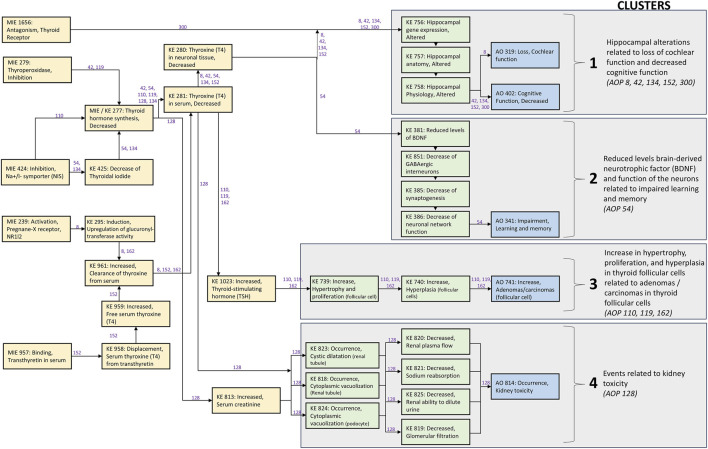
Network of thyroid system related AOPs with mammalian evidence including AOPs 8, 42, 54, 110, 119, 128, 134, 152, 162, 300. Early events (often related to the thyroid system) are displayed by yellow boxes; late events (often more specific for the adverse outcome) are displayed by green boxes, and adverse outcomes are displayed by blue boxes. The numbers in purple indicate the AOP IDs of the AOPs where the key event relationships (KERs) are described. The network can be clustered into four based on the KERs, late events and AOs (grey boxes).

All identified thyroid-related AOPs and their species applicability are provided in Supplementary Table S1, and detailed MIE, KE, and AO information for mammalian-relevant AOPs is provided in Supplementary Table S2. These AOPs served as the basis for the network presented in Figure 1. Non-mammalian AOPs (e.g., amphibian metamorphosis, swim bladder inflation) were recorded but excluded from biomarker evaluation due to limited direct human relevance.

### 2.2 AOP network development and clustering

Mammalian-relevant AOPs were organized into a network to visualize overlapping pathway events and biological connectivity (Figure 1). Manual clustering was performed based on shared late KEs and AOs, consistent with Haigis et al. (Haigis et al., 2023). This approach allowed grouping of AOPs with common downstream pathways to facilitate the subsequent identification of EBMs aligned to specific neurodevelopmental, endocrine, and renal outcomes. The resulting clustered network (Figure 1) informed the selection of late KEs and AOs for targeted biomarker identification.

### 2.3 Extraction of measurement approaches from AOP-Wiki

For each MIE and KE, all measurement methods described in AOP-Wiki were extracted, including molecular, cellular, tissue-level, and functional readouts. Each method was evaluated for its suitability for human epidemiological studies, considering:

- biological matrix availability (blood, urine, saliva, cerebrospinal fluid, tissue)
- feasibility and scalability in population-based research
- degree of invasiveness
- existing human evidence

Measures requiring invasive sampling (e.g., brain tissue histology) were noted as mechanistically informative but not feasible for routine epidemiological use.

### 2.4 Literature search for candidate effect biomarkers

Where AOP-Wiki did not identify feasible human measurement options, we performed targeted PubMed searches. Search terms combined biomarker-related keywords with KE-specific biological terms and applied filters for human or translational evidence, English language, and publication years 2018–2023.

**FIGURE 2:**
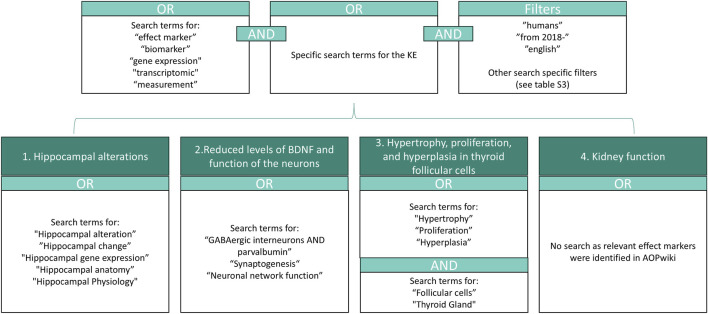
Illustration of the search strategy in PubMed for MIE/KE without relevant measurement methods in AOP-wiki. The specific search strings are given in Supplementary Table S3.

The search workflow is illustrated in Figure 2, and full search strings are provided in Supplementary Table S3. Titles and abstracts were screened to identify studies reporting EBMs aligned to the late KEs and AOs.

### 2.5 Evaluation of biomarker applicability for human studies

Candidate EBMs were then evaluated based on their relevance and potential for use in human studies. Specifically, we considered: a) their mechanistic alignment with the corresponding AOP KEs and AOs, b) availability of measurable endpoints in human biological matrices, and c) feasibility for implementation in epidemiological settings, including scalability in population-based research. When human data was already available, we reviewed this evidence to assess applicability and maturity. Biomarkers supported mainly by experimental studies were noted as promising candidates that will require additional validation before widespread use in human populations. Markers that rely on highly invasive sampling, such as cerebrospinal fluid or post-mortem brain tissue, were not considered feasible for routine human biomonitoring and are presented separately in Supplementary Table S4, as they remain valuable from a mechanistic research perspective.

### 2.6 Reporting and synthesis

The results are organized according to the four mechanistic clusters identified in the AOP network (Figure 1). For each cluster, we describe the relevant biological context, the associated KEs and AOs, and the EBMs identified through our screening process. Where applicable, we highlight EBMs that are already measurable in human biological matrices and have been used in epidemiological or clinical studies, as well as those that appear promising but currently lack sufficient validation in human populations. We also note instances where no suitable biomarker could be identified, reflecting areas where further development and translational research are needed. Summary tables (Tables 1–4) present an overview of biomarker applicability across clusters, while detailed extraction sheets are provided in Supplementary Tables S1–S4.

**TABLE 1:** Possible effect biomarkers for hippocampal alterations (gene expression, anatomy, and physiology) (Cluster 1 in Figure 1).

Effect marker	Matrix	Measurement methods	Comments/Recommended for human epidemiological studies (why, why not)	References
Hippocampal grading using SNIPE	Brain in intact human body	MRI, memory and cognition assess (WMS, MMSE or CDR)	Promising EBM, however, for epidemiological studies the EBM may not be very useful, as the MRI measurement is very inconvenient and time-consuming	Morrison et al. (2023)
BDNF and TrkB gene expression	Postmortem human CA1 pyramidal neurons and regional hippocampal dissections	PCR and hybridization	Promising EBM, if the effects in the brain can be found in non-/low invasive matrices as well	Ginsberg et al. (2019)
QPCTL, APOE, and ERCC2 gene expression	Postmortem human hippocampus samples	Whole genome sequencing and RNA sequencing	Possible future EBM if the altered gene expression can be found in non/low invasive matrices, and associations with the AOs (cochlear function and cognitive function) can be confirmed in heathy individuals	Liu et al. (2021)
ATL2 gene expression	Postmortem human brain samples	Real-time qPCR	Possible future EBM if the altered gene expression can be found in non/low invasive matrices, and associations with the AOs (cochlear function and cognitive function) can be confirmed in heathy individuals	Han et al. (2021)
GAS6 expression	Postmortem human brain samples	*In situ* hybridization of hippocampus (dentate gyrus) tissue	Possible future EBM if the altered gene expression can be found non/low invasive matrices, and associations with the AOs (cochlear function and cognitive function) can be confirmed in heathy individuals	Reemst et al. (2022)
Clock gene expression (Bmal1, Bmal2, Cry1, Cry2, Per1, Per2, and Rev-erbα)	Postmortem mice hippocampus samples	Real-time qPCR	Possible future EBM, if validated in humans, and if the altered gene expression can be found non/low invasive matrices	Fusilier et al. (2021)
Fez1, Fez2, Bcap31 and Kmt2a gene expression	Postmortem mice hippocampus samples	qPCR and RNA-sequencing	The effect is found in a heart failure mouse model, and it needs to be investigated if the same effect is seen in other models with cognitive impairment	Islam et al. (2021)
circRNA cdh9 and Trpc6 expression	Postmortem mice samples of hippocampus, cerebellum, prefrontal cortex, and amygdala	Real-time qPCR Sanger sequencing	Possible future EBM, if validated in humans, if the altered gene expression can be found non/low invasive matrices, and if associations with the AOs (cochlear function and cognitive function) can be confirmed in heathy individuals	Gasparini et al. (2020)

Abbreviations: APOE, Apolipoprotein E; ATL2, Atlastin GTPase, 2; BDNF, brain-derived neurotrophic factor; BMAL1, Basic Helix-Loop-Helix ARNT, Like 1; BMAL2, Basic Helix-Loop-Helix ARNT, Like 2; CA1, Cornu Ammonis 1; cdh9, cadherin 9; CDR, clinical dementia rating; **circRNA**, Circular RNA; **cry1**, cryptochrome circadian regulator 1; **cry2**, cryptochrome circadian regulator 2; EBM, effect biomarker; **ERCC2**, excision repair cross-complementation group 2; **Fez1**, Fasciculation and Elongation Protein Zeta 1; **Fez2**, Fasciculation and Elongation Protein Zeta 2; **GAS6**, Growth arrest-specific 6; **KMT2A**, Lysine Methyltransferase 2A; **MMSE**, mini mental status examination; **MRI**, magnetic resonance imaging; **PCR**, polymerase chain reaction; **Per1**, period circadian regulator 1; **Per2**, period circadian regulator 2; **qPCR**, quantitative polymerase chain reaction; **QPCTL**, Glutaminyl-Peptide Cyclotransferase Like; Rev-erbα, nuclear receptor subfamily 1 group D member 1; **SNIPE**, scoring by nonlocal image patch estimator; **TrkB**, Tropomyosin receptor kinase B; **Trpc6**, transient receptor potential cation channel subfamily C member 6; **WMS**, wechsler memory scale.

## 3 Results

A total of 32 AOPs related to thyroid hormone pathways were identified in the AOP-wiki (Supplementary Table S1), and for 11 AOPs the taxonomic applicability was mammal (human or rodent). However, one AOP (402) did not include any events at the time of extraction. The 10 AOPs included 5 unique adverse outcomes (AOs) and 32 unique molecular initiating events (MIEs)/key events (KEs) (Supplementary Tables S2a, b). The network of the included AOPs is given in Figure 1. Of the 21 excluded non-mammalian AOPs, many focused on reduced anterior swim bladder inflation (n = 5) and altered amphibian metamorphosis (n = 11), not being relevant for human epidemiological studies (Supplementary Table S1).

As seen from the network (Figure 1), the early events are often related to the thyroid hormone system and included in several AOPs, whereas the late events are more specific to the AOs. Thus, even though the early events may be detectable in epidemiological studies before the later events, they may not be specific to the AOs. This supported the focus on late events and AOs for identifying effect biomarkers (EBMs) suitable for human studies.

Several reviews and guidelines are already available for the measurement of thyroid hormones in blood (D'Aurizio et al., 2023; Van Uytfanghe et al., 2023). Therefore, we primarily focused our search on EBMs for the more specific late events but suggest including some EBMs related to thyroid disruption, such as serum thyroid-stimulating hormone (TSH) and thyroxine (T4), in epidemiological studies to support interpretation in relation to upstream endocrine disruption.

Mapping of the 10 mammalian-relevant AOPs resulted in four mechanistic clusters (Figure 1):

Cluster 1: Hippocampal alterations related to loss of cochlear function and decreased cognitive function.

Cluster 2: Reduced levels brain-derived neurotrophic factor (BDNF) and function of the neurons related to impaired learning and memory.

Cluster 3: Increase in hypertrophy, proliferation, and hyperplasia in thyroid follicular cells related to adenomas/carcinomas in thyroid follicular cells.

Cluster 4: Events related to kidney toxicity.

### 3.1 Hippocampal alterations related to loss of cochlear function and decreased cognitive function

Five AOPs (8, 42, 134, 152, 300) with similar KEs on alterations of the hippocampus were identified (Figure 1; Cluster 1). The hippocampal KEs were altered hippocampal gene expression (KE 756), altered hippocampal anatomy (KE 757), and altered hippocampal physiology (KE 758). The five AOPs had two common AOs: loss of cochlear function (AO 319) and decreased cognitive function (AO 402).

We found human relevant measurement methods being described in AOP-wiki, however, the methods were generally not applicable for epidemiological studies as they required brain tissue, e.g., to measure altered gene expression in the hippocampus (Zhang et al., 2002). Some non-invasive, but demanding methods were described in AOP-wiki, such as electroencephalography and structural and functional magnetic resonance imaging. These techniques have been used in several human studies to assess associations between thyroid hormone levels and hippocampal volume/function and cognitive function (Smith and Jonides, 1997; Willoughby et al., 2014; Wheeler et al., 2015; Gilbert et al., 1995). Even though the techniques are well accepted, they are not feasible for larger epidemiological studies due to the requirement of extensive equipment and procedures.

Thus, we searched PubMed for potential EBMs for hippocampal alterations. Of the 56 studies, 8 studies (Morrison et al., 2023; Ginsberg et al., 2019; Liu et al., 2021; Han et al., 2021; Reemst et al., 2022; Fusilier et al., 2021; Islam et al., 2021; Gasparini et al., 2020) were published in 2018 or later and included relevant information on possible EBMs (Table 1). Even though two AOs (decrease cognitive function (AO 402) and loss of cochlear function (AO 319)) were included in the 5 AOPs relevant to this search (Figure 1), all 8 included studies focused on decreased cognitive function and especially Alzheimer’s disease, reflecting the available human evidence base for hippocampal dysfunction.

One study (Morrison et al., 2023) measured the hippocampal grading in humans with magnetic resonance imaging using scoring by Nonlocal Image Patch Estimator (SNIPE) (Table 1). They were able to accurately classify people with normal cognitive function from those with cognitive impairment and Alzheimer’s disease. Even though the tool could be promising for detecting early/mild cognitive impairment in a clinical setting, using magnetic resonance imaging in larger epidemiological studies may be too inconvenient and logistically challenging (Morrison et al., 2023).

The remaining studies measured gene expression levels in postmortem brain/hippocampus tissue, in either humans (Ginsberg et al., 2019; Liu et al., 2021; Han et al., 2021; Reemst et al., 2022) or mice (Fusilier et al., 2021; Islam et al., 2021; Gasparini et al., 2020) (Table 1). Before the possible EBMs can be used in epidemiological studies, they need to be validated in non-/low invasive human matrices such as blood, urine, or saliva.

One of the most promising EBMs identified is the brain-derived neurotrophic factor (BDNF), which has been identified previously as an EBM for neurological outcomes/behavioural function (Rodríguez-Carrillo et al., 2023a; Mustieles et al., 2022). It has already been verified in several epidemiological studies showing that BDNF can be measured in serum, plasma, and urine. High serum BDNF level has also recently been associated with decreased risk of poststroke cognitive impairment after 3 months (Chang et al., 2024). However, to our knowledge, there are no studies in human investigating if the BDNF level in blood and urine is correlated with the BDNF level in the hippocampus, however it is likely, as studies have shown positive correlations between blood and hippocampal BDNF levels in animal models (Klein et al., 2011).

Many of the included studies utilized samples from Alzheimer’s patients (Morrison et al., 2023; Ginsberg et al., 2019; Liu et al., 2021; Han et al., 2021) or from mouse models for Alzheimer’s disease (Fusilier et al., 2021) (Table 1). Alzheimer’s is known for its impact on the hippocampus. It's important to note that some of the EBMs might only emerge after Alzheimer’s disease has become evident. Therefore, these biomarkers may not be effective in early detection of cognitive impairment in epidemiological studies. Some thyroid hormones (free triiodthyronin (fT3) and total triiodthyronin (T3)) have been associated with Alzheimer’s, whereas no associations were seen for thyroid-stimulating hormone (TSH) or thyroxine (T4) in a meta-analysis (Dolatshahi et al., 2023).

### 3.2 Reduced levels brain-derived neurotrophic factor (BDNF) and function of the neurons related to impaired learning and memory

One AOP (54) on impaired learning and memory was identified (Figure 1; Cluster 2). The AOP includes 1 MIE and 9 KEs, and is reviewed and endorsed. We have focused our search on EBMs on the four late KEs: 1. reduced levels of BDNF (KE 381), 2. decrease of GABAergic interneurons (KE 851), 3. decrease of synaptogenesis (KE 385), and 4. decrease of neuronal network function (KE 386).

The KEs are generally well described in AOP-wiki, and the description also includes information on measurement methods. AOP-wiki includes measurement methods for BDNF applicable for epidemiological studies, and BDNF can be measured by available commercial ELISA kits in whole blood, serum, plasma, platelets, and urine (Trajkovska et al., 2007). Furthermore, the EBM has been validated as a marker for neurological outcomes/behavioural function in epidemiological studies (Mustieles et al., 2022; Rodríguez-Carrillo et al., 2023b).

For the remaining KEs, the measurement methods in AOP-wiki were less applicable for epidemiological studies, including for instance immunohistochemical staining on brain tissue and different types of electroencephalography measurements. Therefore, we searched PubMed on relevant novel EBMs for the remaining three late KEs. Twenty articles (Woodworth et al., 2018; Kivimäki et al., 2021; Mullins et al., 2021; Rübsamen et al., 2022; Cortese et al., 2020; Kantrowitz et al., 2021; McClure et al., 2022; Dyrba et al., 2018; Richard et al., 2023; Dong et al., 2023; Kaar et al., 2019; López-Cerdán et al., 2022; Christiansen et al., 2018; Logan et al., 2020; Fagan et al., 2021; Ng et al., 2021; Blennow et al., 2019; Fabbri et al., 2018; Morphett et al., 2024; Gillespie et al., 2024) published in 2018 or later included relevant information on possible EBMs (Table 2). Of the evaluated studies, 8 studies (Woodworth et al., 2018; Kivimäki et al., 2021; Mullins et al., 2021; Rübsamen et al., 2022; Cortese et al., 2020; Kantrowitz et al., 2021; McClure et al., 2022; Dyrba et al., 2018) included potential future EBMs that could be used for human epidemiological studies.

**TABLE 2:** Possible effect biomarkers for neuron function related to impaired learning and memory (Cluster 2 in Figure 1).

Effect marker	Matrix	Measurement methods	Comments/Recommended for human epidemiological studies (why, why not)	References
BDNF	Whole blood, serum, plasma, platelets, and urine	ELISA	Recommended for human epidemiological studies	Trajkovska et al. (2007), Mustieles et al. (2022), Rodríguez-Carrillo et al. (2023b)
Urinary VEGF and its receptor (VEGFR1), MMP2, MMP9, NGAL, Lipocalin 2, and the MMP9/NGAL complex	Human urine	Total protein: Bradford method Specific proteins: ELISA.	Possible future EBM and recommended for human epidemiological studies, and if associations with the AO (cognitive function) can be confirmed in heathy individuals	Woodworth et al. (2018)
SPD, SLIT2, HXK2, CHSTC, AMD, and NCF-1	Human plasma	SomaScan version 4 assay	Recommended for human epidemiological studies, if associations with the AO (cognitive function) can be confirmed in heathy individuals	Kivimäki et al. (2021)
Gene (genomic and expression), such as HTR6, MCHR1, DCLK3 and FURIN	Human brain and whole blood sample	GWAS and TWAS	Possible future EBM if the altered gene expression can be found in non/low invasive matrices, and associations with the AOs (cochlear function and cognitive function) can be confirmed in heathy individuals	Mullins et al. (2021)
Cerebrospinal fluid and blood biomarker proteins (14-3-3β, 14–3-3γ, S100B, t-tau)	Human cerebrospinal fluid and blood	Proteins: Western blot and ELISA.	Possible future EBM and recommended for human epidemiological studies, however it is not clear from the publication if the specific biomarkers were measured in cerebrospinal fluid or blood	Rübsamen et al. (2022)
Vit D	Human serum	ELISA	Possible future EBM combined with other EBMs, due to non specificity and seasonal variation	Cortese et al. (2020)
Glu, Glx, and GABA	Brain imaging of human brain	^1^H MRS	Possible EBM, however, for epidemiological studies the EBM may not be very useful, as the ^1^H MRS measurement is very inconvenient and time-consuming	Kantrowitz et al. (2021)
Hippocampal neurochemical profile (e.g Cr, PCr, Glc, Tau)	Brain imaging of rat hippocampus	^1^H MRS	Possible EBM, if validated in humans. Furthermore, for larger epidemiological studies the EBM may not be very useful, as the ^1^H MRS measurement is very inconvenient and time-consuming	McClure et al. (2022)
Gene expression of Insr, Glut8, Parp1, and Nfkb	Rat hippocampus brain tissue	Real-time qPCR	Possible future effect, if validated in humans, and if the altered gene expression can be found non/low invasive matrices	
Mean amyloid load, glucose metabolism, and gray matter volume	Brain imaging of human brain	Imaging technics: PET, MRI and the additional use of Multimodal Markov random field models	Recommended for human epidemiological studies, however for larger studies the EBM may not be useful due to the inconvenience and logistically challenging with brain imaging	Dyrba et al. (2018)

Abbreviations: ^1^H MRS, proton magnetic resonance spectroscopy; AO, adverse outcome; **AMD**, peptidyl-glycine α-amidating monooxygenase; BDNF, brain-derived neurotrophic factor; **CHSTC**; carbohydrate sulfotransferase 12; Cr, creatine; DCLK3, doublecortin like kinase 3; ELISA, Enzyme-linked immunosorbent assay; EBM, effect biomarker; FURIN, furin; GABA, gamma-aminobutyric acid; Glc, Glucose; Glx, glutamate; GWAS, Genome-wide association study; HTR6, 5-hydroxytryptamine receptor 6; HXK2, hexokinase 2; Insr, Insulin receptor; MCHR1, melanin concentrating hormone receptor 1; MMP-2, matrix metalloproteinase- 2; MMP9, matrix metalloproteinase- 9; NCF-1, neutrophil cytosol factor 1; Nfkb, nuclear factor kappa B; NGAL, neutrophil gelatinaseassociated lipocalin; Parp1, poly (ADP-ribose) polymerase 1; PCr, phosphocreatine; PET, positron emission tomography; qPCR, quantitative polymerase chain reaction; SLIT2, slit homologue 2 protein; SPD, Protein levels of protein D; Tau, taurine; t-tau, total taurine; TWAS, transcriptome-wide association study; VEGF, vascular endothelial growth factor; VEGFR1, Vascular endothelial growth factor receptor 1; Vit D, 25-hydroxyvitamin-D (25(OH)D).

One study (Woodworth et al., 2018) measured urinary protein levels of metalloproteinase-9 (MMP9) and MMP9/Neutrophil gelatinase-associated lipocalin (NGAL) complex and found that higher levels may enhance neuronal damage in the brainstem nuclei and enhance plasticity of sensorimotor regions in human patients with urological chronic pelvic pain syndrome (Table 2). Further validation of the associations with the KEs and the AOs in healthy individuals are, however, needed before implementation of the EBMs in epidemiological studies.

Four studies used blood as a matrix and measured protein levels (Kivimäki et al., 2021; Rübsamen et al., 2022), gene expressions (Mullins et al., 2021), and vitamin D (Cortese et al., 2020) (Table 2). The studies were conducted in people with diseases (multiple sclerosis, bipolar disorder, Alzheimer’s disease, sporadic Creutzfeldt-Jakob disease) (Kivimäki et al., 2021; Mullins et al., 2021; Rübsamen et al., 2022; Cortese et al., 2020). Thus, in all cases further validation of the associations with the KEs and the AOs in healthy human individuals are needed before implementation of the EBMs in epidemiological studies.

Three studies (Kantrowitz et al., 2021; McClure et al., 2022; Dyrba et al., 2018), with potential future EBMs, used brain imaging of rodent and human brains to evaluate the neuron function (Table 2). These methods can be used in human studies, however, for larger epidemiological studies the inconvenience and logistic challenges may be difficult to overcome.

The remaining 12 studies (Richard et al., 2023; Dong et al., 2023; Kaar et al., 2019; López-Cerdán et al., 2022; Christiansen et al., 2018; Logan et al., 2020; Fagan et al., 2021; Ng et al., 2021; Blennow et al., 2019; Fabbri et al., 2018; Morphett et al., 2024; Gillespie et al., 2024) without potential EBMs recommended for human epidemiological studies mainly used invasive samples (e.g., brain tissue and cerebrospinal fluid) as a matrix. These studies may increase the understanding of the mechanisms involved in the pathways towards AOs and disease (Supplementary Table S4).

### 3.3 Increase in hypertrophy, proliferation, and hyperplasia in thyroid follicular cells related to adenomas/carcinomas in thyroid follicular cells

Three AOPs (110, 119, 162) with increase in adenomas/carcinomas in thyroid follicular cells as AO (AO 741) were identified, and included several identical KEs (Figure 1; Cluster 3). We have focused our search on EBMs for the two late KEs: increase in hypertrophy and proliferation (KE 739) and increase in hyperplasia (KE 740) in follicular cells.

The description of the two KEs in AOP-wiki is very limited and does not include any information on measurement methods. Therefore, we searched PubMed, and of 115 hits, 13 articles were published in 2018 or later and included relevant information on possible EBMs for increase in hypertrophy, proliferation, and hyperplasia thyroid and/or adenomas/carcinomas in thyroid follicular cells (Zhang P. et al., 2020; Ameziane et al., 2019; Halsall and Oddy, 2021; Yan et al., 2021; Wang et al., 2020; Bertoni et al., 2021; Pang and Yang, 2021; Wang et al., 2018; Zhou et al., 2018; Hong et al., 2023; Bertoni et al., 2019; Mostoufi-Moab et al., 2018; Qu et al., 2020).

The information on possible EBMs in the 13 included studies are listed in Table 3.

**TABLE 3:** Possible effect biomarkers for increase in hypertrophy, proliferation, and hyperplasia in follicular cells and/or adenomas/carcinomas in follicular cells (Cluster 3 in Figure 1).

Effect marker	Matrix	Measurement methods	Comments/Recommended for human epidemiological studies (why, why not)	References
IL-34	Human serum	ELISA	Potential EBM, however, related to several other diseases and is not specifically related to adenomas/carcinomas in follicular cell	Zhang X. et al. (2020)
Oxidative stress (TAS, TOS, ratio of TOS to TAS)	Human serum/Whole Blood	Commercial kits; Electronic paramagnetic resonance	Potential EBM, however, related to several other diseases and is not specifically related to adenomas/carcinomas in follicular cell	Ameziane et al. (2019)
Reverse T3	Human serum	Mass-spectrometric methods	Potential EBM, however, related to several other diseases and is not specifically related to adenomas/carcinomas in follicular cell	Halsall and Oddy (2021)
ARHGAP36 gene expression	Primary human papillary thyroid carcinoma; metastasis lymph node; precancerous tissue	Immunohistochemistry staining; Real-time qPCR	Possible future EBM, however, the study was conducted on thyroid tissue and lymph node, a matrix not normally available in large epidemiological studies	Yan et al. (2021)
S100A12 gene expression	Human papillary thyroid carcinoma tissue and adjacent non-cancerous tissue	Immunohistochemistry staining; Western blot assay	Possible future EBM, however, the study was conducted on thyroid tissue and lymph node, a matrix not normally available in large epidemiological studies	Wang et al. (2020)
GPER1 gene expression	Human thyroid tumour versus matched non-cancerous thyroid tissues	Real-time-qPCR; Immunohistochemistry	Possible future EBM, however, the study was conducted on thyroid tissue and lymph node, a matrix not normally available in large epidemiological studies	Bertoni et al. (2021)
DUXAP8	Human tumour tissues and the adjacent normal tissues	qPCR; Western blot	Possible future EBM, however, the study was conducted on thyroid tissue and lymph node, a matrix not normally available in large epidemiological studies	Pang and Yang (2021)
miRNA “hsa-miR-200a-5p”	Human papillary benign thyroid tumours with papillary hyperplasia versus thyroid carcinomas patients	qPCR; Immunohistochemistry	Possible future EBM, however, the study was conducted on thyroid tissue and lymph node, a matrix not normally available in large epidemiological studies	Wang et al. (2018)
circRNA “has_circ_0008274”	Human papillary thyroid cancer tissue and matched adjacent tissues	qPCR; Western blot	Possible future EBM, however, the study was conducted on thyroid tissue and lymph node, a matrix not normally available in large epidemiological studies	Zhou et al. (2018)
TSP1 gene expression and slicing variants	Human thyroid carcinoma tissue versus adjacent non-malignant thyroid tissue	Antibody array, Real-time-PCR	Possible future EBM, however, the study was conducted on thyroid tissue and lymph node, a matrix not normally available in large epidemiological studies	Hong et al. (2023)
AMPase activity and NT5E expression	Human papillary thyroid carcinoma tissue versus adjacent non-malignant thyroid tissue	Enzymatic activities of ectonucleotidases; qPCR	Possible future EBM, however, the study was conducted on thyroid tissue and lymph node, a matrix not normally available in large epidemiological studies	Bertoni et al. (2019)
Variations in RAS, RET/PTC rearrangement and BRAF	Human tissue of different stages/types of thyroid follicular carcinomas	qPCR	The somatic variations may serve as a future EBM, if the variations could be detected in blood samples/circulating tumour cells	Mostoufi-Moab et al. (2018)
Somatic variations in RET (RTK/RAS pathway)	Primary human thyroid tumour versus matched noncancerous thyroid tissues	Whole-exome sequencing on DNA extracted from tissue	The somatic variations may serve as a future EBM, if the variations could be detected in blood samples/circulating tumour cells	Qu et al. (2020)

Abbreviations: AMPase, Adenosine 5′-monophosphatase; ARHGAP36, Rho GTPase, activating protein 36; BRAF, B-Raf proto-oncogene, serine/threonine kinase; circRNA, Circular RNA; DUXAP8, double homeobox A pseudogene 8; ELISA, Enzyme-linked immunosorbent assay; EBM, effect biomarker; GPER1, G protein-coupled estrogen receptor 1; IL-34, Interleukin 34; miRNA, micro RNA; NT5E, 5′-nucleotidase ecto; PCR, polymerase chain reaction; qPCR, quantitative polymerase chain reaction; S100A12, S100 calcium binding protein A12; T3, triiodothyronine; TAS, total antioxidant status; TOS, total oxidant status; TSP1, tumor suppressor region 1.

A potential EBM in blood is interleukin-34 (IL-34), which is increased in both tumour tissue and serum of papillary thyroid cancer patients compared with age-matched controls (Zhang P. et al., 2020) (Table 3). The expression of serum IL-34 was also significantly associated with tumour size, tumour stage, and lymph node metastasis (Zhang P. et al., 2020). IL-34 has also been found to play a role in many other cancer types, such as hepatocarcinoma, osteosarcoma, multiple myeloma, colon cancer, and lung cancer (Franzè et al., 2020). Furthermore, IL-34 has been linked to several other disease types including autoimmune disorders, infections, neurological disorders and metabolic diseases, indicating that it is not very specific as EBM (Baghdadi et al., 2018).

Two other EBMs measurable in serum, oxidative stress (Ameziane et al., 2019) and reverse triiodothyronine (rT3) (Halsall and Oddy, 2021), have been identified in the literature (Table 3). They are, however, too unspecific to be EBMs alone on increase in hypertrophy, proliferation, hyperplasia and/or adenomas/carcinomas in thyroid follicular cells but might serve as EBMs in combination with other markers.

Eight studies (Yan et al., 2021; Wang et al., 2020; Bertoni et al., 2021; Pang and Yang, 2021; Wang et al., 2018; Zhou et al., 2018; Hong et al., 2023; Bertoni et al., 2019) have identified differences in expression of genes, microRNA and circularRNA, as well as slicing variants and adenosine monophosphatase (AMPase) activity between tumour tissue and matched adjacent or normal non-cancerous tissue (Table 3). These markers may serve as future EBMs, if the differences can be detected in blood samples or other non-/low invasive matrixes which can be collected in larger epidemiological studies. Development and validation of these suggested EBMs need further studies.

Two studies (Mostoufi-Moab et al., 2018; Qu et al., 2020) found somatic and germline DNA variations present in the thyroid tumours compared to non-cancerous tissues (Table 3). The somatic variation might serve as future EBMs if detectable in blood samples. Both studies found somatic variations related to the receptor tyrosine kinase (RTK)/RAS pathway (Mostoufi-Moab et al., 2018; Qu et al., 2020), which could be relevant to investigate more in relation to EBMs, as well as the hippo pathway identified by Qu et al. (Qu et al., 2020).

### 3.4 Events related to kidney toxicity

One AOP (128) on kidney toxicity was identified as a thyroid-related AOP (Figure 1; Cluster 4). The AOP includes two events related to thyroid hormone balance (MIE 277 and KE 281), increased serum creatinine (KE 813) and seven late KEs related to kidney function including cystic dilatation (KE 823), cytoplasmic vacuolization (KE 818 and KE 824), decrease renal plasma flow (KE 820), sodium reabsorption (KE 821), ability to dilute urine (KE 825), and glomerular filtration (KE 819).

The descriptions of the AOP and KEs in AOP-wiki are very limited and do not include any information on measurement methods. However, several EBMs of kidney function have already been identified and reviewed by others (Zare et al., 2021; Rodríguez-Carrillo et al., 2023a; Lopez-Giacoman and Madero, 2015; Fassett et al., 2011; Wasung et al., 2015; Kidney Disease: Improving Global Outcomes KDIGO, 2013; Younes-Ibrahim and Younes-Ibrahim, 2022). Therefore, we have not conducted additional systematic literature searches, but Table 4 comprises the most relevant information on the already identified EBMs. In Table 4, we have also indicated the potential relationship between the EBMs and the KEs. However, establishing definitive connections is challenging due to the limited information available in AOP-wiki regarding the AOP and KEs.

**TABLE 4:** Effect biomarkers identified for kidney toxicity (Cluster 4 in Figure 1).

Effect marker	Matrix	Measurement method	Comments/Recommended for human epidemiological studies (why, why not)	References
Creatinine	Human serum/urine	Kinetic alkaline picrate assay (Jaffe method)/ELISA	Recommended and commonly used as a measure of kidney function	Lopez-Giacoman and Madero (2015), Wasung et al. (2015), Younes-Ibrahim and Younes-Ibrahim (2022), Zhang X. et al. (2020)
α1-microglobulin (A1-MG)	Human plasma/urine	ELISA/nephelometric immunoassay	Recommended as an early EBM of tubular disorders	Penders and Delanghe (2004), Mustieles et al. (2018)
Retinol-binding protein 4 (RBP4)	Human plasma/urine	Mass-spectrometry/ELISA	Recommended and is the most sensitive functional biomarker of proximal tubule	Lopez-Giacoman and Madero (2015), Lapsley et al. (1998), Ratajczyk et al. (2022)
Albumin	Human urine	Immunological assays/turbidimetric assays	Recommended and provides a more specific and sensitive measure of changes in glomerular permeability than urinary total protein	(Kidney Disease: Improving Global Outcomes KDIGO, 2013; Younes-Ibrahim and Younes-Ibrahim, 2022)
N-acetyl-β-D glucosaminidase (NAG)	Human serum/urine	ELISA/Fluorometric assay	Recommended and part of the FDA qualified biomarker panel for kidney tubular injury (U.S. Food and Drug Administration, 2020). Early EBM of tubular damage useful for human biomonitoring purposes	Kim et al. (2015)
KIM-1 (kidney injury molecule-1)	Human serum/urine	ELISA/laminar-flow dipstick assay	Recommended and part of the FDA qualified biomarker panel for kidney tubular injury (U.S. Food and Drug Administration, 2020). Early biomarker for proximal tubular damage	(Lopez-Giacoman and Madero, 2015; Wasung et al., 2015; Yin and Wang, 2016)
Neutrophil gelatinase-associated lipocalin (NGAL)	Human serum/plasma/urine	ELISA/chemiluminescence immunoassay	Recommended and part of the FDA qualified biomarker panel for kidney tubular injury (U.S. Food and Drug Administration, 2020). Marker for proximal and distal tubule disorders and an early marker for acute kidney injury as well as chronic kidney damage	(Lopez-Giacoman and Madero, 2015; Wasung et al., 2015; Younes-Ibrahim and Younes-Ibrahim, 2022; Turgut et al., 2020)
Cystatin C (CysC)	Human serum/urine	ELISA/nephelometric immunoassay/immunoturbidimetry	Recommended and part of the FDA qualified biomarker panel for kidney tubular injury (U.S. Food and Drug Administration, 2020). Early marker of acute kidney injury and early kidney dysfunction, as well as a marker of chronic kidney disease	(Lopez-Giacoman and Madero, 2015; Wasung et al., 2015; Younes-Ibrahim and Younes-Ibrahim, 2022; Spanaus et al., 2010)
β2-microglobulin (B2-MG)	Human plasma/serum/urine	ELISA/nepherometric immunoassay	Recommended as a marker of GFR and an early EBM of tubular damage useful for human biomonitoring purposes	(Lopez-Giacoman and Madero, 2015; Kim et al., 2015)
Clusterin (CLU)	Human serum/urine	ELISA	Recommended and part of the FDA qualified biomarker panel for kidney tubular injury (U.S. Food and Drug Administration, 2020). Urinary level changes are specific to kidney injury	(Dieterle et al., 2010; Musiał et al., 2020)
Osteopontin (OPN)	Human serum/urine	ELISA	Recommended and part of the FDA qualified biomarker panel for kidney tubular injury (U.S. Food and Drug Administration, 2020), an also suggested EBM of chronic kidney disease	(de Borst and Carrero, 2023; Sinha et al., 2023)
Fractional excretion of solutes (FeS)	Human plasma and urine	Measurement of solutes, such as sodium in both plasma and urine to calculate the fractional excretion	Recommended as a simple EBM for tubular function, which could be related to both acute and chronic kidney injury	Younes-Ibrahim and Younes-Ibrahim (2022)

Abbreviations: A1-MG, α1-microglobulin; B2-MG, β2-microglobulin; **CLU**, clusterin; **CysC**, cystatin-C; **ELISA**, Enzyme-linked immunosorbent assay; EBM, effect biomarker; **FDA**, U.S., food and drug administration; **FeS**, fractional excretion of solutes; **GFR**, glomerular filtration rate; KIM-1, kidney injury molecule-1; **NAG**, N-acetyl-beta-d-glucosaminidase; **NGAL**, neutrophil gelatinase-associated lipocalin; **OPN**, osteopontin; **RBP4**, Retinol-binding protein 4.

Utilizing a panel of EBMs can provide a more comprehensive assessment of kidney damage and functional changes and help to identify early signs of kidney injury that may not be detectable with traditional markers alone. The U.S. Food and Drug Administration (FDA) has in 2018 assessed a panel of six markers for kidney tubular injury in clinical Phase 1 trials for drugs (U.S. Food and Drug Administration, 2020): clusterin (CLU), cystatin-C (CysC), kidney injury molecule-1 (KIM-1), N-acetyl-beta-d-glucosaminidase (NAG), neutrophil gelatinase-associated lipocalin (NGAL), and osteopontin (OPN). The U.S. FDA determined that the Composite Measure composed of these six urinary biomarkers was qualified as a “safety composite biomarker panel to be used in conjunction with traditional measures to aid in the detection of kidney tubular injury in phase 1 trials in healthy volunteers when there is an a priori concern that a drug may cause renal tubular injury in humans” (U.S. Food and Drug Administration, 2020).

## 4 Discussion

This study presents a comprehensive search for effect biomarkers (EBMs) for thyroid hormone-related AOPs, using the AOP framework to identify potential EBMs for use in epidemiological research. We identified potential EBMs for most of the included AOPs, some have already been used epidemiologically and are validated, whereas others are novel and need further development and validation before complete implementation in epidemiological studies. This illustrates both the translational potential of AOP-aligned EBMs and current gaps that require continued research.

The most promising EBM identified was BDNF for the AOPs on both “Hippocampal alterations related to loss of cochlear function and decreased cognitive function” (cluster 1) and “Reduced levels of BDNF and function of the neurons related to impaired learning and memory” (cluster 2) (Figure 1). For the AOPs on “Increase in hypertrophy, proliferation, and hyperplasia in thyroid follicular cells related to adenomas/carcinomas in thyroid follicular cells” (Figure 1, cluster 3), we did not identify any single EBM with high specificity, but IL-34, oxidative stress and gene expression of several genes were related to the key events (KEs) and adverse outcomes (AOs). For the AOP on kidney toxicity (Figure 1, cluster 4), a panel of markers including Retinol-binding protein 4 (RBP4), KIM-1, CysC, NAG and NGAL was identified and has demonstrated utility in epidemiological studies as well as regulatory qualification.

### 4.1 Interpretation of AOP-derived mechanistic clusters

#### 4.1.1 BDNF as effect biomarker for decreased cognitive function and impairment of learning and memory

The gene expression, protein level, and/or methylation status of the BDNF gene were identified as promising EBMs for both cluster 1 and 2 in the network of thyroid system related AOPs with mammalian evidence (Figure 1). BDNF is a neurotrophins which plays a role in survival and differentiation of neurons during development and regulates synaptic transmission (excitatory and inhibitory) and activity-dependent plasticity in adulthood (Lu et al., 2014). The importance of BDNF in cognitive function and memory is well established (Lu et al., 2014). The two AOP clusters (Babić et al., 2021; Burch, 2019) include three AOs (“loss of cochlear function”, “decreased cognitive function”, and “impairment of learning and memory”), and it is therefore not possible to distinguish between these AOs using BDNF as an EBM. However, the AOPs are indeed closely related as they share many of the early KEs and some of the AOs are very similar. Reduced level of BDNF is a specific KE for AOP 54, indicating a strong relevance in that context. The serum BDNF level has also been related to other neurological disorders and diseases such as attention deficit hyperactivity disorder, major depressive disorder, and autism spectrum disorder (Rodríguez-Carrillo et al., 2023b). BDNF levels in peripheral blood are considered as an optimal estimate for the brain BDNF concentration, as it can cross the blood-brain barrier (Koven and Collins, 2014). It is also possible to measure in urine, where the fasting morning urine BDNF levels correlate positively with serum levels (Olivas-Martinez et al., 2023). However, urinary BDNF may not be the optimal EBM for cognitive function, as it has also been associated with bladder issues (overactive bladder, enuresis) and visceral chronic pain related conditions (Rodríguez-Carrillo et al., 2023b). Overall, BDNF is a feasible and biologically relevant EBM, though further work is needed to confirm correspondence between peripheral and central levels in humans.

#### 4.1.2 Effect biomarkers for thyroid cancer

Potential but more unspecific EBMs (IL-34, oxidative stress, etc.) were identified for proliferation and/or adenomas/carcinomas arising in follicular thyroid cells. The included AOPs (110, 119, 162) specifically address pathways with thyroid follicular cell hypertrophy and/or hyperplasia leading to follicular cell adenomas/carcinomas. The AOPs are based on rodent evidence and may be less relevant for humans, where the cancer risk associated with thyroid follicular hyperplasia (goiter) is low (Huisinga et al., 2020). This was pointed out by a panel of experts from different sectors, including pharmaceutical and agrochemical industries, contract research organizations, academic research laboratories, and regulatory affairs for drug, food, and chemical safety gathered by the European Society of Toxicologic Pathology in 2018 (Huisinga et al., 2020). However, thyroid cancer is considered a relevant AO of exposure to endocrine-disrupting chemicals (Macedo et al., 2023). In a recent review, Macedo et al. (2023) identified 30 human studies on endocrine-disrupting chemicals and thyroid cancer, of which approximately half reported significant associations.

The most accepted risk factor for developing human thyroid cancer in general is radiation exposure during childhood and in some cases a family history of thyroid cancer or iodine deficiency (Huisinga et al., 2020; Kruger et al., 2022). In addition, several types of endocrine disrupting chemicals have been associated to thyroid cancer in epidemiological studies, including dioxins, heavy metals, persistent organic pollutants, pesticides, and phthalates (Macedo et al., 2023; Kruger et al., 2022), underscoring the need for mechanistic understanding and tools tailored to human thyroid carcinogenesis. Thus, developing relevant AOPs for human thyroid cancers may be important to improve the understanding of mechanisms and effects of environmental exposures, as well as developing more relevant EBMs for epidemiological studies.

We also found one study (Costante and Meringolo, 2020) suggesting calcitonin as a good EBM for human C-cell cancer (accounts for 1%–5% of all thyroid cancer cases (Ramos et al., 2025)). Calcitonin has the advantage that it can be measured in blood with commercially available immunoassays. Calcitonin is a hormone secreted by the C-cells of the thyroid gland. It plays a crucial role in regulating calcium levels in the blood by inhibiting bone resorption though reducing osteoclast activity and motility, as well as helps to protect against hypercalcemia (Felsenfeld and Levine, 2015). Serum calcitonin has not been linked with many other diseases and could be a relatively specific marker of C-cell thyroid cancer; however, some studies have found an association with bronchogenic cancer as well (Silva et al., 1976; Silva et al., 1979). Thus, while promising for C-cell neoplasia, calcitonin does not yet represent a broadly applicable thyroid tumour EBM.

#### 4.1.3 Effect biomarker for kidney toxicity

EBMs of kidney function are well investigated, and several markers for different kidney diseases have been identified and validated. The present review did therefore not include a systematic search on EBMs related to AOP 128 on kidney toxicity (Figure 1, cluster 4), however, the most common EBMs from other reviews and the U.S. FDA were presented and described (Zare et al., 2021; Rodríguez-Carrillo et al., 2023a; Lopez-Giacoman and Madero, 2015; Fassett et al., 2011; Wasung et al., 2015; Kidney Disease: Improving Global Outcomes KDIGO, 2013; U.S. Food and Drug Administration, 2020) (Table 4). The U.S. FDA approved a panel of six urinary EBMs for kidney tubular injury in clinical Phase 1 trials for drugs (U.S. Food and Drug Administration, 2020), the EBM panel may not only be relevant in clinical trials of drugs but could also be used in epidemiological studies. However, a validation of the sensitivity and specificity in epidemiological studies is needed. It is common to include a panel of EBMs in studies on chemical exposure and kidney toxicity (Ortega-Romero et al., 2023; Pérez-Herrera et al., 2019; Saylor et al., 2022; Wallin et al., 2014). Although individual kidney EBMs are considered relatively specific for kidney injury, they reflect different biological processes and compartments within the kidney and therefore differ in sensitivity depending on exposure type, timing, and population characteristics. For instance, in a study on inorganic element exposure and kidney function in children (Ortega-Romero et al., 2023), Ortega-Romero et al. measured urinary levels of neutrophil gelatinase-associated lipocalin (NGAL), cystatin-C (CysC), osteopontin (OPN), clusterin (CLU), α1-microglobulin (A1-MG), and kidney injury molecule-1(KIM-1). They observed that the level of inorganic elements (fluoride, vanadium, arsenic, potassium, and sodium) correlated positively with some of the kidney injury EBMs (KIM-1, NGAL, α1-MG) (Ortega-Romero et al., 2023). This highlights the relevance of including a panel of EBMs, as they may have different sensitivity according to the setting and study population and the need for harmonized sampling and analytical protocols to support comparability across cohorts.

Rather than serving as stand-alone indicators, kidney EBMs are therefore best interpreted jointly, as a composite reflecting multiple dimensions of kidney stress and injury. Including multiple EBMs can also improve the comparability and reproducibility of results across studies, which is crucial for establishing robust scientific conclusions. Most of the suggested kidney EBMs have the advantage that they are measurable in urine samples, a non-invasive matrix that is highly recommended in human biomonitoring and epidemiological studies. However, the type of urine sample (spot, morning, or 24-h) and adjustment for urinary dilution (creatinine/specific gravity) is debated (Helmersson-Karlqvist et al., 2016). Even though spot samples are most convenient, they are variable, whereas morning samples are more stable. The use of 24-h samples in larger epidemiological studies is limited due to the inconvenience, logistical challenges, and lower compliance. Standardized recommendations are needed to harmonize collection and adjustment methods, enhancing study comparability and reliability.

### 4.2 Application of AOP-based effect biomarkers in epidemiological studies of chemical exposures

Integrating AOPs into epidemiological research provides a mechanistic framework for linking chemical exposures (environmental pollutants or drugs) to health outcomes. Many widely used chemicals engage the molecular initiating events (MIEs) within the presented AOP network (Figure 1). Here, we summarize some of the broadly prevalent exposures that can act through these events, with emphasis on three MIEs present in multiple AOPs: decreased thyroid hormone synthesis, inhibition of thyroid peroxidase (TPO), and inhibition of the sodium/iodide symporter (NIS).

Decreased thyroid hormone synthesis (MIE/KE 277) is part of all four identified AOP clusters and included in six of the AOPs (Figure 1; Supplementary Tables S2a, b). It is one of the most essential events for THSD, and generally included as a KE, but for AOP 128 it is given as the MIE. Deceased thyroid hormone synthesis cannot be measured directly but is often measured indirectly by TPO and NIS inhibition or by T4 serum levels.

Inhibition of TPO (MIE 279) is the MIE of two included AOPs (42 and 119) leading to decreased cognitive function and follicular cell adenomas and carcinomas, respectively. TPO is the enzyme responsible for the *de novo* synthesis of thyroid hormones, and its inhibition can disrupt systemic thyroid hormone homeostasis. High-throughput screening of over 1000 chemicals from the ToxCast library identified more than 300 putative TPO inhibitors, spanning pharmaceuticals, pesticides, industrial compounds, and consumer product ingredients (Paul et al., 2016). Among these, bisphenols and phthalates stand out due to their widespread use in plastics, food packaging, and personal care products, resulting in ubiquitous exposure across the general population (Gerofke et al., 2024; Govarts et al., 2023). Observational evidence suggests associations between exposure to these compounds and altered circulating thyroid hormone levels in humans (Milczarek-Banach et al., 2020; Kim et al., 2019). Given the prevalence of bisphenols and phthalates in everyday environments and their demonstrated potential to interfere with thyroid hormone synthesis via TPO inhibition, further epidemiological studies to validate the effects in humans are warranted. Focusing on the AOs of the included AOPs (decreased cognitive function and follicular cell adenomas/carcinomas) and aim to validate the identified EBMs.

Inhibition of NIS (MIE 424) leads to decreased cognitive function, impaired learning and memory, and follicular cell adenomas and carcinomas in the included AOP (54, 110, and 134). NIS is essential for iodide uptake into thyroid follicular cells, the initial step for thyroid hormone synthesis. Recent high-throughput screening of over 1000 environmental chemicals identified 273 compounds with significant inhibition of NIS-mediated iodide uptake, highlighting the broad chemical space with potential to impair thyroid hormone synthesis (Wang et al., 2019). Several of the identified NIS inhibitors are common exposures in the general population, such as perchlorate, thiocyanate and nitrate that occur in drinking water, foods and tobacco smoke and have been associated with altered circulating thyroid hormone levels in population studies, with stronger effects in women of lower iodine status (Blount et al., 2006; Blount et al., 2007; Bruce et al., 2013; King et al., 2023). Several per- and polyfluoroalkyl substances (PFAS) have also been identified as potential NIS inhibitors in humans (Stoker et al., 2023). Population exposure to PFAS is well documented worldwide, including Europe, the United States, Asia and the Arctic (Richterová et al., 2023; Botelho et al., 2025; Kookana et al., 2025; Bonefeld-Jørgensen et al., 2023), underscoring their epidemiologic relevance. Accordingly, epidemiological studies should evaluate associations between NIS inhibitors, such as perchlorate, thiocyanate, nitrate and PFAS, and health effects via AOP-aligned EBMs.

### 4.3 Developmental status, taxonomic applicability, and future perspectives and needs

The 11 AOPs included in this study are at various stages of development, highlighting the evolving nature of thyroid hormone-related research within the AOP framework. Two AOPs (42 (cluster 1) and 54 (cluster 2)) have received endorsement (Supplementary Table S1), indicating a higher level of scientific consensus and validation, and provide a strong foundation for regulatory applications. The 9 AOPs (within cluster 1, 3, and 4 and AOP 402 without any events) are under development with several requiring additional empirical evidence to strengthen key event relationships (KERs) and improve predictive utility for epidemiological studies (Supplementary Table S1).

The developmental stage of the AOP and associated events directly affect the reliability of the identified EBMs. EBMs linked to well-developed AOPs and validated KEs are more reliable and applicable for human studies, while those associated with less-developed pathways require further validation before they can be widely used in population-based research. The variability in the developmental status of the AOPs highlights the need for continued research to enhance their robustness and regulatory acceptance. Future efforts should prioritize the validation of key events and measurement methods, ensuring their applicability for human biomonitoring and risk assessment purposes.

All AOPs with a mammal (human and rodent) applicability domain were included in the search of EBMs for epidemiological studies. Five AOPs (42, 54, 128, 134, 300) explicitly state human relevance, while six (8, 110, 119, 152, 162, 402) had rat and/or mouse as the taxonomic applicability domain (Supplementary Table S1). This inclusive approach ensures broader coverage, capturing a wide range of potential EBMs. Although animal models provide valuable mechanistic insights, human relevance must be validated, especially for AOPs such as those describing follicular cell adenomas and carcinomas, which may not be directly applicable to humans.

Thyroid disruption can profoundly affect human health, particularly neurodevelopment during critical windows such as the foetal and early postnatal periods. Despite the comprehensive nature of current AOPs for THSD, there may still be endpoints that are not fully covered (Noyes et al., 2019). Current AOPs primarily focus on well-characterized MIEs and KEs, but there are gaps in understanding the full spectrum of thyroid disruption effects (Noyes et al., 2019).

The use of EBMs in epidemiological studies offers significant advantages in THSD. EBMs can provide early indications of biological changes, facilitate timely intervention and improve risk assessment. They can also enhance the specificity and sensitivity of epidemiological studies, allowing for more accurate detection of thyroid-related health effects. Specific EBM for the thyroid hormone system, such as serum TSH and thyroxine (T4), are already well-established in clinical practice for monitoring thyroid function (D'Aurizio et al., 2023; Van Uytfanghe et al., 2023). However, novel EBMs, including the neurotoxicity marker (BDNF) and kidney markers (e.g., NGAL and KIM-1), have shown promise in linking thyroid disruption to more specific health outcomes. When individual EBMs exhibit limited specificity, their interpretative value in epidemiological studies can be strengthened through the combined use of multiple EBMs capturing complementary biological processes within an AOP. Such panel-based approaches can improve biological plausibility and robustness, particularly for complex outcomes where multiple mechanistically linked pathways contribute to the adverse outcome.

By integrating EBMs into the AOP framework, we can bridge the gap between mechanistic toxicology (*in-vitro* and animal studies) and epidemiological research, ultimately leading to better understanding and management of THSD’s impact on human health. This approach not only enhances the predictive power of AOPs but also supports the development of targeted interventions and regulatory policies to mitigate the adverse effects of thyroid disruptors. However, validation of the EBMs is needed to ensure the validity of the results. Thus, while the use of BDNF and kidney markers seem to be implemented and validated in several epidemiological studies, further work is needed to validate other novel EBMs (Tables 1–4).

### 4.4 Prioritization of effect biomarkers for epidemiological studies

To support practical implementation of AOP-aligned EBMs in human studies, we prioritized the identified EBMs according to their biological specificity, feasibility in commonly used human biological matrices, strength of human evidence, and remaining validation needs (Table 5). This provides a translational link between the mechanistic evidence captured in the AOPs and the requirements of population-based research and regulation.

**TABLE 5:** Prioritization of AOP-aligned EBMs for human studies.

Biomarker/Panel	AOP Cluster(s)	Feasibility	Specificity	Human evidence	Development need	Priority
BDNF (serum/plasma)	1–2	High	Moderate	Moderate	Moderate	High
BDNF (urine)	1–2	High	Low–Moderate	Low–Moderate	High	Moderate
CLU, CysC, KIM-1, NAG, NGAL, OPN	4	High	High	High	Low–Moderate	High
Calcitonin	3	High	Moderate–High	Low	Moderate	Moderate
IL-34 & oxidative stress markers	3	Moderate	Low–Moderate	Low	High	Low
TSH, Free T4	All	High	High	High	Low	Context markers

Abbreviations: AOP, adverse outcome pathway; BDNF, brain-derived neurotrophic factor; **CLU**, clusterin; **CysC**, cystatin-C; KIM-1, kidney injury molecule-1; **NAG**, N-acetyl-beta-d-glucosaminidase; **NGAL**, neutrophil gelatinase-associated lipocalin; **OPN**, osteopontin; IL-34, Interleukin 34; TSH, thyroid-stimulating hormone; **T4**, thyroxine.

Brain-derived neurotrophic factor (BDNF) and the kidney injury biomarkers (clusterin (CLU), cystatin-C (CysC), kidney injury molecule-1 (KIM-1), N-acetyl-beta-d-glucosaminidase (NAG), neutrophil gelatinase-associated lipocalin (NGAL), osteopontin (OPN)) emerged as high-priority candidates, as they are biologically relevant, measurable in minimally invasive matrices, and increasingly supported by human data. Calcitonin is promising for C-cell neoplasia but has limited epidemiological applicability due to the rarity of this outcome. Interleukin-34 (IL-34) and oxidative stress markers are mechanistically relevant but require further specificity and validation before broad use.

Overall, this prioritization highlights a set of EBMs that can be deployed in epidemiological research now, as well as those requiring targeted development to enable regulatory uptake and refinement of thyroid-related AOPs.

## 5 Conclusion

In 10 thyroid hormone system related AOPs with mammalian relevance, we have searched for human epidemiological relevant EBMs. We identified brain-derived neurotrophic factor (BDNF) as the most promising EBM for the AOPs leading to decreased cognitive function and impaired learning and memory (AOP 42, 54, 134, 152, and 300), while a panel of EBMs were identified for the kidney toxicity (AOP 128). These EBMs have already been implemented in several human epidemiological studies. However, specific EBMs for AOP 8 on loss of cochlear function needed further work. The AOPs on thyroid follicular adenomas and carcinomas (AOP 110, 119, and 162) had limited human relevance, but a subset of potential EBMs for other human thyroid cancers was identified. Overall, this study highlights the relevance and potential of using EBMs based on AOPs in epidemiological studies to enhance the understanding of the impact by THSD on human health. By aligning mechanistic toxicology with human observational data, AOP-based EBMs provide a structured and biologically grounded path toward strengthening causal inference, improving risk assessment, and informing regulatory decision-making. Continued refinement and validation of EBMs, particularly for pathways with limited human evidence, will further support their integration into public health research and chemical regulation.

## Glossary

18[F]FDG: [18F]flurodeoxyglucose
^**1**^ **H MRS**: Proton magnetic resonance spectroscopy
AMD: peptidyl-glycine α-amidating monooxygenase
AMP: Adenosine Monophosphate
AMPase: Adenosine 5′-monophosphatase
AO: adverse outcome
AOP: Adverse Outcome Pathway
APOE: Apolipoprotein E
ARHGAP36: Rho GTPase activating protein 36
ATL2: Atlastin GTPase 2
B2-MG: β2-microglobulin
BCAP31: B-Cell Receptor Associated Protein 31
BDNF: brain-derived neurotrophic factor
BMAL1: Basic Helix-Loop-Helix ARNT Like 1
BMAL2: Basic Helix-Loop-Helix ARNT Like 2
BRAF: B-Raf proto-oncogene, serine/threonine kinase
CA1: Cornu Ammonis 1
cdh9: cadherin 9
CDR: Clinical Dementia Rating
CHSTC: carbohydrate sulfotransferase 12
circRNA: Circular RNA
CLU: Clusterin
Cr: creatine
cry1: cryptochrome circadian regulator 1
cry2: cryptochrome circadian regulator 2
CysC: Cystatin C
DCLK3: doublecortin like kinase 3
DNA: Deoxyribonucleic Acid
DUXAP8: double homeobox A pseudogene 8
ELISA: Enzyme-linked immunosorbent assay
EBM: Effect biomarker
FDA: U.S. Food and Drug Administration
FeS: Fractional excretion of solutes
Fez1: Fasciculation and Elongation Protein Zeta 1
Fez2: Fasciculation and Elongation Protein Zeta 2
FURIN: furin
GABA: gamma-aminobutyric acid
GAS6: Growth arrest-specific 6
GFR: glomerular filtration rate
Glc: Glucose
Glu: glutamate
Glut8: solute carrier family 2 member 12 (known as SLC2A12)
Glx: glutamine
GPER1: G protein-coupled estrogen receptor 1
GWAS: Genome-wide association study
HBM: human biomonitoring
HBM4EU: Human biomonitoring for Europe
HTR6: 5-hydroxytryptamine receptor 6
HXK2: hexokinase 2
IL-34: Interleukin 34
Insr: Insulin receptor
ITGA9: integrin subunit alpha 9
KCC2: Potassium Chloride Cotransporter 2
KE: key event
KER: key event relationship
KIM-1: kidney injury molecule-1
KMT2A: Lysine Methyltransferase 2A
MCHR1: melanin concentrating hormone receptor 1
MIE: molecular initiating event
miRNA: micro RNA
MMP-2: matrix metalloproteinase- 2
MMP9: metalloproteinase-9
MMSE: Mini Mental Status Examination
MRI: Magnetic resonance imaging
mRNA: messenger ribonucleic Acid
NAG: N-acetyl-β-D glucosaminidase
NCF-1: neutrophil cytosol factor 1
Nfkb: nuclear factor kappa B
NGAL: Neutrophil gelatinase-associated lipocalin
NT5E: 5′-nucleotidase ecto
OPN: Osteopontin
PARC: Partnership for the Assessment of Risks from Chemicals
Parp1: poly(ADP-ribose) polymerase 1
PCr: phosphocreatine
PCR: Polymerase chain reaction
Per1: period circadian regulator 1
Per2: period circadian regulator 2
PET: positron emission tomography
PFAS: Per- and polyfluoroalkyl substance
qPCR: Quantitative Polymerase chain reaction
QPCTL: Glutaminyl-Peptide Cyclotransferase Like
RAS: receptor tyrosine kinase
RBP4: Retinol-binding protein 4
Rev-erbα: nuclear receptor subfamily 1 group D member 1
RNA: Ribonucleic Acid
rT3: Reverse Triiodothyronine
S100A12: S100 calcium binding protein A12
S100B: S100 calcium-binding protein B
SNIPE: Scoring by Nonlocal Image Patch Estimator
SPD: Protein levels of protein D
T3: triiodthyronin
T4: thyroxine
TAS: Total antioxidant status
Tau: taurine
THSD: Thyroid hormone system disruption
TOS: Total oxidant status
TPO: thyroid peroxidase
TrkB: Tropomyosin receptor kinase B
Trpc6: transient receptor potential cation channel subfamily C member 6
TSH: thyroid-stimulating hormone
TSP1: tumor suppressor region 1
t-tau: total taurine
TWAS: transcriptome-wide association study
VEGF: vascular endothelial growth factor
VEGFR1: Vascular endothelial growth factor receptor 1
Vit D: 25-hydroxyvitamin-D (25(OH)D)
WMS: Wechsler Memory Scale
