# Supplementary material for: Epidemiological relevant effect biomarkers for thyroid hormone system related adverse outcome pathways: a literature review
Source: Front Pharmacol. 2026 Mar 4;17:1760820. doi: 10.3389/fphar.2026.1760820 (PMC12996058; doi:10.3389/fphar.2026.1760820)
Supplement: Supplementary file 2 [file Table4.pdf]

Table S4 Studies with not recommended epidemiological effect biomarkers for neuron function related to impaired learning and memory (Cluster 2 in Figure 1)

Effect marker	Matrix	Measurement methods	Comments / Recommended for human epidemiological studies (Why, why not)	Reference
Parvalbumin (PV)	Brain tissue (rodents/human postmortem)	Immunohistochemistry, RNA-seq, Microarray	Invasive methods that require brain samples, not recommended for human epidemiological studies.	(43)
GAD67 (GAD1) and GAD65 (GAD2)		Immunohistochemistry, RNA-seq		
Calretinin		Immunohistochemistry		
Calbindin		Immunohistochemistry		
KCC2 protein	Brain tissue (rodents)	Western blot, Immunohistochemistry		
Perineuronal nets (PNNs), including PTPσ, Otx2, ErbB4, and GAG	Human brain tissue		Invasive methods that require brain samples, not recommended for human epidemiological studies.	(44)
Parvalbumin cell density and parvalbumin mRNA levels	Human brain tissue	Immunocytochemistry of parvalbumin cell density in prefrontal cortex.<br>qPCR parvalbumin mRNA levels in prefrontal regions.	Invasive method as it requires brain samples, not recommended for human epidemiological studies.	(45)
Transcriptomic data by sex	Human brain tissues: frontal cortex, striatum, and substantia nigra	Transcriptomics analysis on brain tissue. The current study used public available data from 8 studies.	Invasive method as it requires brain samples, not recommended for human epidemiological studies.	(46)
NPY expressed in GABAergic and calretinin-immunoreactive (-ir) amacrine cells	Human and porcine retina	Immunohistochemistry, confocal microscopy and 3D reconstructions	Not recommended for human epidemiological studies at this stage as retina is needed for measurement. Unknown if NPY is linked to GABAergic neurons in the brain, and to the adverse outcome (impaired learning and memory)	(47)
Expression of specific neuronal markers such as MAP2 and PAX6, as well as synaptic markers like Syn1.	In vitro human cerebral organoids	Immunostaining	Not recommended for human epidemiological studies.<br>Comparing biomarkers from cerebral organoids with foetal and adult brain samples strengthens the model's credibility, making it a valuable tool for exploring brain development, function, and disorders, as well as synaptogenesis.	(48)
Synapse structure		Electron microscopy on fixed cerebral organoids		
mRNA expression of MAP2, OCT4, S100B, SCN1A, SCN2A, SCN3A, SCN8A, SCN9A, and SMA		Real time qPCR		
Action Potential and Different Channel Activities		Electrophysiology analysis assessed action potential and channel activities, including recording glutamate, NMDA, and GABA currents, which are indicative of synaptic transmission.		
Amyloid-β (Aβ40, Aβ42), pTau181, neuronal/axonal/synaptic injury (t-tau, VILIP-1,	Human cerebrospinal fluid	Immunoassays of β40, Aβ42, t-Tau and pTau181.	Invasive method requires cerebrospinal fluid, not recommended for human epidemiological studies.	(49)

NfL, SNAP-25), and astro-gliosis/neuroinflammation (YKL-40)		SNAP-25 and VILIP-1 were measured using Single Molecule Counting technology using antibodies.<br>YKL-40 was measured with ELISA.<br>Also, cognitive test and genetic tests were carried out.	Persons with Down syndrome often develop Alzheimer disease, and studying the two groups may enhance the understanding of disease mechanisms.	
Aβ1–42, t-tau and ptau181	Human cerebrospinal fluid; Human brain imaging	Aβ1–42, t-tau and p-tau181 levels by immunoassay; [18F]FDG PET; Questionnaire (NPI-Q)	Invasive method with cerebrospinal fluid, not recommended for human epidemiological studies. The study due not include healthy individuals for comparison but include cognitively intact carriers of Dominantly inherited Alzheimer's disease in mutation carriers and non-carriers.	(50)
Neurogranin, t-tau, NFL and 14-3-3 protein	Human cerebrospinal fluid	ELISA	Invasive method with cerebrospinal fluid, not recommended for human epidemiological.	(51)
Genetic variations in ITGA9 and NRXN3	Human blood	Analysis of SNP	Not recommended for epidemiological studies. Germline variations could serve as predictors of risk, but not as EBM as they will be present from birth.	(52)
Perineuronal nets (PNNs)	Brain tissue from rodents; Brain imaging of rodents	Immunohistochemical or immunofluorescent staining with lectin WFA and VVA. Quantification of PNNs is typically determined through 1) number counts of PNNs, 2) intensity of PNNs, and 3) colocalization of PNNs and parvalbumin neurons.	Not recommended for human epidemiological studies, as the measurement requires incisions of the brain or intracranial injections. The review only included rodent studies; thus, the findings must be confirmed in humans.	(53)
Gene expression of Dlx1, Dlx2, Nkx2.1, Lhx6, and Arx	Brain tissue from rodents	RT-PCR	Not recommended for human epidemiological studies, as the measurement requires brain tissue. The review only included rodent studies; thus, the findings must be confirmed in humans.	(54)

Abbreviations: [18F]FDG: [18F]flurodeoxyglucose; **ARX:** Aristaless Related Homeobox; **Dlx1:** distal-less homeobox 1; **Dlx2:** distal-less homeobox 2; **ELISA:** Enzyme-linked immunosorbent assay; **EBM:** Effect biomarker; **ErbB4:** erb-b2 receptor tyrosine kinase 4; **GABA:** gamma-aminobutyric acid; **GAD65:** Glutamate Decarboxylase 65 (GAD2 gene); **GAD67:** Glutamate Decarboxylase 67 (GAD1 gene); **GAG:** Glycosaminoglycan; **ITGA9:** integrin subunit alpha 9; **KCC2:** Potassium Chloride Cotransporter 2; **Lhx6:** LIM Homeobox 6; **MAP2:** microtubule associated protein 2; **NFL:** neurofilament light chain; **Nkx2.1:** NK2 homeobox 1; **NMDA**: *N*-methyl-d-aspartate **NPI-Q:** Neuropsychiatric Inventory-Questionnaire; **NPY:** Neuropeptide Y; **NRXN3:** neurexin 3; **OCT4:** organic cation/carnitine transporter4 i; **Otx2:** orthodenticle homeo-box 2; **PNN:** Perineuronal net; **pTau181:** phosphorylated tau-related processes; **PTPσ:** protein tyrosine phosphatase σ; **PV:** Parvalbumin; **qPCR:** Quantitative Polymerase Chain Reaction; **RT-PCR:** Real-time Polymerase Chain Reaction; **RT-QuIC:** Real-time quaking-induced conversion;
