# Supplementary material for: Epidemiological relevant effect biomarkers for thyroid hormone system related adverse outcome pathways: a literature review
Source: Front Pharmacol. 2026 Mar 4;17:1760820. doi: 10.3389/fphar.2026.1760820 (PMC12996058; doi:10.3389/fphar.2026.1760820)
Supplement: Supplementary file 3 [file Table1.pdf]

Table S1: Thyroid related AOPs identified in AOPwiki (Month, Year) sorted by species relevance.

Color code: Evidence from human studies | Evidence from rodent studies | Evidence from non-mammal studies

AOP Wiki Id	Title	Author status	OECD status	Species	Life stage	MIE	AO (or organismal KE)	Via (central KE)	Stressors
**42**	Inhibition of Thyroperoxidase and Subsequent Adverse Neurodevelopmental Outcomes in Mammals	Open for citation & comment	WPHA/WNT Endorsed	Human, Rat, Mouse	During brain development, Development	TPO inhibition	decreased cognitive function	Thyroxine (T4) in serum, Decreased	Methimazole, Propylthiouracil
**54**	Inhibition of Na+/I- symporter (NIS) leads to learning and memory impairment	Open for citation & comment	WPHA/WNT Endorsed	Human, Rat	Fetal, Perinatal, During brain development	NIS inhibition	learning and memory impairment	Thyroxine (T4) in serum, Decreased	Perchlorate, Nitrate, Thiocyanate, Dysidenin, Aryltrifluoroborates
**128**	Kidney dysfunction by decreased thyroid hormone	Under development: Not open for comment. Do not cite	Under Development	Human, Rat	1 to < 3 months, Adult	decreased TH synthesis	kidney dysfunction	Thyroxine (T4) in serum, Decreased	
**134**	Sodium Iodide Symporter (NIS) Inhibition and Subsequent Adverse Neurodevelopmental Outcomes in Mammals	Under Development: Contributions and Comments Welcome		Human, Rat	Perinatal	NIS inhibition	decreased cognitive function	Thyroxine (T4) in serum, Decreased	
**300**	Thyroid Receptor Antagonism and Subsequent Adverse Neurodevelopmental Outcomes in Mammals	Under development: Not open for comment. Do not cite	Under Development	Human, Mouse	During brain development	TR antagonism	decreased cognitive function	not applicable	
**8**	Upregulation of Thyroid Hormone Catabolism via Activation of Hepatic Nuclear Receptors, and Subsequent Adverse Neurodevelopmental Outcomes in Mammals	Open for adoption	Under Development	Rat	Fetal to Parturition, Nursing Child	hepatic nuclear receptors	loss of cochlear function	Thyroxine (T4) in serum, Decreased	PCB
**110**	Inhibition of iodide pump activity leading to follicular cell adenomas and carcinomas (in rat and mouse)	Under Development: Contributions and Comments Welcome		Rat, Mouse	?	NIS inhibition	follicular cell adenoma/carcinoma	Thyroxine (T4) in serum, Decreased	
**119**	Inhibition of thyroid peroxidase leading to follicular cell adenomas and carcinomas (in rat and mouse)	Under Development: Contributions and Comments Welcome		Rat, Mouse	?	TPO inhibition	follicular cell adenoma/carcinoma	Thyroxine (T4) in serum, Decreased	
**152**	Interference with thyroid serum binding protein transthyretin and subsequent adverse human neurodevelopmental toxicity	Under Development: Contributions and Comments Welcome	Under Development	Rat	Development	TTR binding	decreased cognitive function	Thyroxine (T4) in serum, Decreased	Halogenated phenols, Polychlorinated biphenyl, Polychlorinated dibenzodioxins, Polybrominated diphenyl ethers, Isoflavones, Perflourinated chemicals, Phthalates, Tetrabromobisphenol A, Clonixin, Meclofenamic acid, 2,6-dinitro-p-cresol, Triclopyr, 2,2',4,4'-Tetrahydroxybenzophenone
**162**	Enhanced hepatic clearance of thyroid hormones leading to thyroid follicular cell adenomas and carcinomas in the rat and mouse	Under Development: Contributions and Comments Welcome		Rat, Mouse		hepatic nuclear receptors	follicular cell adenoma/carcinoma	Thyroxine (T4) in serum, Decreased	Phenobarbital, thiazopyr, Pyrethrins and Pyrethroids
**402**	Thyroid peroxidase (TPO) inhibition leads to periventricular heterotopia formation in the developing rat brain	Under development: Not open for comment. Do not cite		Rat?	Development?	TPO inhibition	Heterotopia formation	not specified	
**155**	Deiodinase 2 inhibition leading to increased mortality via reduced posterior swim bladder inflation	Under Development: Contributions and Comments Welcome	WPHA/WNT Endorsed	Fethead minnow, Zebrafish	Embryo	DIO2 inhibition	reduced swim bladder inflation	Triiodothyronine (T3), Decreased	
**156**	Deiodinase 2 inhibition leading to increased mortality via reduced anterior swim bladder inflation	Under Development: Contributions and Comments Welcome	WPHA/WNT Endorsed	Fethead minnow, Zebrafish	Larvae	DIO2 inhibition	reduced swim bladder inflation	Triiodothyronine (T3), Decreased	
**157**	Deiodinase 1 inhibition leading to increased mortality via reduced posterior swim bladder inflation	Under Development: Contributions and Comments Welcome	WPHA/WNT Endorsed	Fethead minnow, Zebrafish	Embryo	DIO1 inhibition	reduced swim bladder inflation	Triiodothyronine (T3), Decreased	
**158**	Deiodinase 1 inhibition leading to increased mortality via reduced anterior swim bladder inflation	Under Development: Contributions and Comments Welcome	WPHA/WNT Endorsed	Fethead minnow, Zebrafish	Larvae	DIO1 inhibition	reduced swim bladder inflation	Triiodothyronine (T3), Decreased	
**159**	Thyroperoxidase inhibition leading to increased mortality via reduced anterior swim bladder inflation	Under Development: Contributions and Comments Welcome	WPHA/WNT Endorsed	Fethead minnow, Zebrafish	Larvae	TPO inhibition	reduced swim bladder inflation	Thyroxine (T4) in serum, Decreased Triiodothyronine (T3), Decreased	Methimazole, Mercaptobenzothiazole, Propylthiouracil
**175**	Thyroperoxidase inhibition leading to altered amphibian metamorphosis	Under Development: Contributions and Comments Welcome		African clawed frog	Development	TPO inhibition	altered amphibian metamorphosis	Thyroxine (T4) in serum, Decreased	Methimazole, Propylthiouracil, Mercaptobenzothiazole, 2,2',4,4'-Tetrahydroxybenzophenone
**176**	Sodium Iodide Symporter (NIS) Inhibition leading to altered amphibian metamorphosis	Under Development: Contributions and Comments Welcome		African clawed frog	Development	NIS inhibition	altered amphibian metamorphosis	Thyroxine (T4) in serum, Decreased	Perchlorate
**188**	Iodotyrosine deiodinase (IYD) inhibition leading to altered amphibian metamorphosis	Under Development: Contributions and Comments Welcome		African clawed frog	Development	IYD inhibition	altered amphibian metamorphosis	Thyroxine (T4) in serum, Decreased	
**189**	Type I iodothyronine deiodinase (DIO1) inhibition leading to altered amphibian metamorphosis	Under Development: Contributions and Comments Welcome		African clawed frog	Development	DIO1 inhibition	altered amphibian metamorphosis	Triiodothyronine (T3), Decreased	
**190**	Type II iodothyronine deiodinase (DIO2) inhibition leading to altered amphibian metamorphosis	Under Development: Contributions and Comments Welcome		African clawed frog	Development	DIO2 inhibition	altered amphibian metamorphosis	Triiodothyronine (T3) in tissues, Decreased Thyroxine (T4), Increased	
**191**	Type III iodotyrosine deiodinase (DIO3) inhibition leading to altered amphibian metamorphosis	Under Development: Contributions and Comments Welcome		African clawed frog	Development	DIO3 inhibition	altered amphibian metamorphosis	Triiodothyronine (T3) in tissues, Increased	
**192**	Pendrin inhibition leading to altered amphibian metamorphosis	Under Development: Contributions and Comments Welcome		African clawed frog	Development	pendrin inhibition	altered amphibian metamorphosis	Thyroxine (T4) in serum, Decreased	
**193**	Dual oxidase (DUOX) inhibition leading to altered amphibian metamorphosis	Under Development: Contributions and Comments Welcome		African clawed frog	Development	DUOX inhibition	altered amphibian metamorphosis	Thyroxine (T4) in serum, Decreased	
**194**	Hepatic nuclear receptor activation leading to altered amphibian metamorphosis	Under Development: Contributions and Comments Welcome		African clawed frog	Development	hepatic nuclear receptors	altered amphibian metamorphosis	Thyroxine (T4) in serum, Decreased	
**271**	Inhibition of thyroid peroxidase leading to impaired fertility in fish	Open for comment. Do not cite	Under Development	Fish	Adult	TPO inhibition	impaired fertility	Thyroid hormone synthesis, Decreased	Propylthiouracil, Methimazole, Ethylene thiourea
**363**	Thyroperoxidase inhibition leading to increased mortality via altered eye structure	Open for citation & comment	EAGMST Under Review	Zebrafish	Embryo, Larvae	TPO inhibition	altered visual function	Thyroxine (T4) in serum, Decreased Triiodothyronine (T3), Decreased	Propylthiouracil, Methimazole
**366**	Competitive binding to thyroid hormone carrier protein transthyretin (TTR) leading to altered amphibian metamorphosis	Under development: Not open for comment. Do not cite		Amphibian	?	TTR binding	altered amphibian metamorphosis	Thyroxine (T4) in serum, Decreased	
**367**	Competitive binding to thyroid hormone carrier protein thyroid binding globulin (TBG) leading to altered amphibian metamorphosis	Under development: Not open for comment. Do not cite		Amphibian	?	TBG binding	altered amphibian metamorphosis	Thyroxine (T4) in serum, Decreased	
**364**	Thyroperoxidase inhibition leading to increased mortality via decreased eye size	Under development: Not open for comment. Do not cite	Under Development	?	?	TPO inhibition	altered visual function	Thyroxine (T4) in serum, Decreased Triiodothyronine (T3), Decreased	
**365**	Thyroperoxidase inhibition leading to increased mortality via altered photoreceptor patterning	Under development: Not open for comment. Do not cite	Under Development	?	?	TPO inhibition	altered visual function	Thyroxine (T4) in serum, Decreased Triiodothyronine (T3), Decreased	
**393**	AOP for thyroid disorder caused by triphenyl phosphate	Under development: Not open for comment. Do not cite		?	?	hepatic TH uptake/transport	Apoptosis	not applicable	Triphenyl phosphate
