# Supplementary material for: Epidemiological relevant effect biomarkers for thyroid hormone system related adverse outcome pathways: a literature review
Source: Front Pharmacol. 2026 Mar 4;17:1760820. doi: 10.3389/fphar.2026.1760820 (PMC12996058; doi:10.3389/fphar.2026.1760820)
Supplement: Supplementary file 4 [file Table3.pdf]

Table S3: Search strings used in the PubMed searches

Search	Date of search	Search string
1. Hippocampal alterations	January 23, 2024	("effect marker"[Title/Abstract] OR "biomarker"[Title/Abstract] OR "gene expression"[Title/Abstract] OR "transcriptomic"[Title/Abstract] OR “measurement”[Title/Abstract]) AND ("Hippocampal alteration"[Title/Abstract] OR "Hippocampal change"[Title/Abstract] OR "Hippocampal gene expression"[Title/Abstract] OR "Hippocampal anatomy"[Title/Abstract] OR "Hippocampal Physiology"[Title/Abstract]) AND (humans[Filter]) AND (2018:3000/12/12[pdat])
2a. GABAergic interneurons	February 27, 2024	(("gabaergic"[All Fields] OR "gabaergics"[All Fields]) AND ("interneuron s"[All Fields] OR "interneuronal"[All Fields] OR "interneuronally"[All Fields] OR "interneurone"[All Fields] OR "interneurones"[All Fields] OR "interneurons"[MeSH Terms] OR "interneurons"[All Fields] OR "interneuron"[All Fields]) AND ("biomarker s"[All Fields] OR "biomarkers"[MeSH Terms] OR "biomarkers"[All Fields] OR "biomarker"[All Fields] OR ("marker"[All Fields] OR "markers"[All Fields]) OR (("effect"[All Fields] OR "effecting"[All Fields] OR "effective"[All Fields] OR "effectively"[All Fields] OR "effectiveness"[All Fields] OR "effectivenesses"[All Fields] OR "effectives"[All Fields] OR "effectivities"[All Fields] OR "effectivity"[All Fields] OR "effects"[All Fields]) AND ("marker"[All Fields] OR "markers"[All Fields])) OR ("parvalbumins"[MeSH Terms] OR "parvalbumins"[All Fields] OR "parvalbumin"[All Fields]))) AND ((booksdocs[Filter] OR casereports[Filter] OR classicalarticle[Filter] OR clinicalstudy[Filter] OR clinicaltrial[Filter] OR clinicaltrialprotocol[Filter] OR clinicaltrialphasei[Filter] OR clinicaltrialphaseii[Filter] OR clinicaltrialphaseiii[Filter] OR clinicaltrialphaseiv[Filter] OR veterinaryclinicaltrial[Filter] OR comparativestudy[Filter] OR controlledclinicaltrial[Filter] OR meta-analysis[Filter] OR observationalstudy[Filter] OR veterinaryobservationalstudy[Filter] OR randomizedcontrolledtrial[Filter]) AND (humans[Filter] OR animal[Filter]) AND (2018:3000/12/12[pdat]) AND (english[Filter]))
2b. Synaptogenesis	February 27, 2024	("synaptogenesis"[All Fields] AND ("biomarker s"[All Fields] OR "biomarkers"[MeSH Terms] OR "biomarkers"[All Fields] OR "biomarker"[All Fields] OR ("marker"[All Fields] OR "markers"[All Fields]) OR (("effect"[All Fields] OR "effecting"[All Fields] OR "effective"[All Fields] OR "effectively"[All Fields] OR "effectiveness"[All Fields] OR "effectivenesses"[All Fields] OR "effectives"[All Fields] OR "effectivities"[All Fields] OR "effectivity"[All Fields] OR "effects"[All Fields]) AND ("marker"[All Fields] OR "markers"[All Fields]))) AND ((booksdocs[Filter] OR casereports[Filter] OR classicalarticle[Filter] OR clinicalstudy[Filter] OR clinicaltrial[Filter] OR clinicaltrialprotocol[Filter] OR clinicaltrialphasei[Filter] OR clinicaltrialphaseii[Filter] OR clinicaltrialphaseiii[Filter] OR clinicaltrialphaseiv[Filter] OR veterinaryclinicaltrial[Filter] OR comparativestudy[Filter] OR controlledclinicaltrial[Filter] OR meta-analysis[Filter] OR observationalstudy[Filter] OR veterinaryobservationalstudy[Filter] OR randomizedcontrolledtrial[Filter]) AND (humans[Filter] OR animal[Filter]) AND (2018:3000/12/12[pdat]) AND (english[Filter]))
2c. Neuronal network function	February 27, 2024	(("neuron s"[All Fields] OR "neuronal"[All Fields] OR "neuronally"[All Fields] OR "neuronals"[All Fields] OR "neurone s"[All Fields] OR "neurones"[All Fields] OR "neuronic"[All Fields] OR "neurons"[MeSH Terms] OR "neurons"[All Fields] OR "neuron"[All Fields] OR "neurone"[All Fields]) AND ("network"[All Fields] OR "network s"[All Fields] OR "networked"[All Fields] OR "networker"[All Fields] OR "networkers"[All Fields] OR "networking"[All Fields] OR "networks"[All Fields]) AND ("functional"[All Fields] OR "functional s"[All Fields] OR "functionalities"[All Fields] OR "functionality"[All Fields] OR "functionalization"[All Fields] OR "functionalizations"[All Fields] OR "functionalize"[All Fields] OR "functionalized"[All Fields] OR "functionalizes"[All Fields] OR "functionalizing"[All Fields] OR "functionally"[All Fields] OR "functionals"[All Fields] OR "functioned"[All Fields] OR "functioning"[All Fields] OR "functionings"[All Fields] OR "functions"[All Fields] OR "physiology"[MeSH Subheading] OR "physiology"[All Fields] OR "function"[All Fields] OR "physiology"[MeSH Terms]) AND ("biomarker s"[All Fields] OR "biomarkers"[MeSH Terms] OR "biomarkers"[All Fields] OR "biomarker"[All Fields] OR ("marker"[All Fields] OR "markers"[All Fields]) OR (("effect"[All Fields] OR "effecting"[All Fields] OR "effective"[All Fields] OR "effectively"[All Fields] OR "effectiveness"[All Fields] OR "effectivenesses"[All Fields] OR "effectives"[All Fields] OR "effectivities"[All Fields] OR "effectivity"[All Fields] OR "effects"[All Fields]) AND ("marker"[All Fields] OR "markers"[All Fields])) OR ("synaptic"[All Fields] OR "synaptical"[All Fields] OR "synaptically"[All Fields])) AND ("epidemiologically"[All Fields] OR "epidemiology"[MeSH Terms] OR "epidemiology"[All Fields] OR "epidemiologic"[All Fields] OR "epidemiological"[All

		Fields])) AND ((booksdocs[Filter] OR casereports[Filter] OR classicalarticle[Filter] OR clinicalstudy[Filter] OR clinicaltrial[Filter] OR clinicaltrialprotocol[Filter] OR clinicaltrialphasei[Filter] OR clinicaltrialphaseii[Filter] OR clinicaltrialphaseiii[Filter] OR clinicaltrialphaseiv[Filter] OR veterinaryclinicaltrial[Filter] OR comparativestudy[Filter] OR controlledclinicaltrial[Filter] OR meta-analysis[Filter] OR observationalstudy[Filter] OR veterinaryobservationalstudy[Filter] OR randomizedcontrolledtrial[Filter]) AND (humans[Filter] OR animal[Filter]) AND (2018:3000/12/12[pdat]) AND (english[Filter]))
3. Hypertrophy, proliferation, and hyperplasia in follicular cells	December 15, 2023	("effect marker"[Title/Abstract] OR "biomarker"[Title/Abstract] OR "gene expression"[Title/Abstract] OR "transcriptomic" [Title/Abstract] OR “measurement”[Title/Abstract]) AND ("Hypertrophy"[Title/Abstract] OR “Proliferation”[Title/Abstract] OR “Hyperplasia”[Title/Abstract]) AND (“Follicular cells”[Title/Abstract] OR "Thyroid Gland"[MeSH]) AND (humans[Filter]) AND (2018:3000/12/12[pdat]) AND (english[Filter]))
